# Novel Intersection Type Recognition for Autonomous Vehicles Using a Multi-Layer Laser Scanner

**DOI:** 10.3390/s16071123

**Published:** 2016-07-20

**Authors:** Jhonghyun An, Baehoon Choi, Kwee-Bo Sim, Euntai Kim

**Affiliations:** 1 School of Electrical and Electronic Engineering, Yonsei University, 50 Seodaemun-gu Sinchon-dong, Seoul 120-743, Korea; jhonghyen@yonsei.ac.kr (J.A.); choibae@yonsei.ac.kr (B.C.); 2 School of Electrical and Electronics Engineering, Chung-Ang University, 84 Heukseok-Ro Dongjak-Gu, Seoul 156-756, Korea; kbsim@cau.ac.kr

**Keywords:** multi-laser scanner, intersections, recognition, local coordinate, occupancy grid map, static map

## Abstract

There are several types of intersections such as merge-roads, diverge-roads, plus-shape intersections and two types of T-shape junctions in urban roads. When an autonomous vehicle encounters new intersections, it is crucial to recognize the types of intersections for safe navigation. In this paper, a novel intersection type recognition method is proposed for an autonomous vehicle using a multi-layer laser scanner. The proposed method consists of two steps: (1) static local coordinate occupancy grid map (SLOGM) building and (2) intersection classification. In the first step, the SLOGM is built relative to the local coordinate using the dynamic binary Bayes filter. In the second step, the SLOGM is used as an attribute for the classification. The proposed method is applied to a real-world environment and its validity is demonstrated through experimentation.

## 1. Introduction

When an autonomous vehicle drives on urban roads, the vehicle encounters a number of traffic intersections and is expected to pass the intersections smoothly all together with other vehicles without causing any trouble. For smooth and safe passing through the urban intersections, it is of crucially importance for the autonomous vehicle to recognize what type of an intersection the current one is. Furthermore, the intersection type recognition is also a valuable cue to the global localization. When the autonomous vehicle has no idea about its location due to the blackout of the GPS or a cold start, one of the possible solutions for the global localization in this situation will be the intersection type recognition proposed herein. For the intersection type recognition, three kinds of sensors are widely used: a camera, radar, and laser scanner.

The use of a camera is popular in the intersection type recognition and several research works have been reported [1,2,3,4]. Mostly, they use gray value gradients to extract the lane markings or roadsides. Admittedly, however, the camera-based intersection type recognition methods have the drawback that they are computationally very expensive and sensitive to the change of luminance and weather condition.

In [5], road boundaries were detected using radar by making the maps of the static environment such as an occupancy grid map. Using a similar method, radar sensors are expected to be used for the intersection type recognition. Furthermore, radar sensors inform the system not only of the position of an obstacle but also its velocity because they use the Doppler Effect. Thus, the moving objects could be easily detected and removed from the grid maps if the radars are used for the intersection type recognition. Unfortunately, the current commercial radar has too low of a resolution to be used as a primary sensor, and it should be used together with a secondary sensor such a laser scanner.

The use of a 3D laser scanner is also popular in the intersection type recognition [6,7,8,9]. In [6,7], Quanwen et al. developed a beam model for the 64-layer scanning lidar and applied it to the intersection type recognition. In [8], Alberto et al. extracted the road curb and navigable surface as features and developed the road geometry recognition system including types of intersections. The system used an artificial neural network (ANN) as a classifier. In [9], Chen et al. proposed a toe-finding algorithm to detect the admissible space and intersection using a 64-layer scanning lidar. Unfortunately, however, the 3D multi-layer laser scanner has some problems: it is economically too expensive to commercialize and the associated algorithm is computationally too expensive to implement in real-time. Also, this sensor ruins the design of the vehicles. Thus, a 2D multi-layer laser scanner with 3 or 4 layers can be a solution to these drawbacks.

The 2D scanner with 3 or 4 layers is cheaper than the full 3D scanner. So, it is widely used for detection such as vehicle or pedestrian [10,11,12]. Furthermore, the 2D multi-layer laser scanner is much more design-friendly than the 3D one since it can be installed and concealed on the frontal bumper. Some research works about the intersection type recognition using the 2D multi-layer laser scanner have been reported [13,14,15]. T. Weiss et al. proposed a road boundary detection algorithm in intersections by using laser scanners. They proposed the imaginary center line concept. These two imaginary center lines detect the occupied cells in grid map to find the boundaries. Unfortunately, the previous intersection type recognition methods using 2D multi-layer laser scanners have some drawbacks: First, the associated algorithms are computationally expensive because they require building a wide global occupancy grid map. Furthermore, the previous works were applied only to simple intersections scenarios such as T-shape junctions.

In this paper, a new method for the intersection type recognition using a multi-layer laser scanner is proposed. When our autonomous vehicle faces an intersection, the proposed method classifies the type of the intersection into six classes: highway with more than two lanes, merge-roads, diverge-roads, plus-shape intersections, and two types of T-shape junctions.

Compared with the previous works [13,14,15], the proposed method builds the occupancy grid map (OGM) not relative to the global coordinate but relative to the local coordinate, more specifically, relative to the ego vehicle coordinates. The local OGM turns to represent the nearby environment from the ego vehicle’s point of view. The local OGM building, however, is not as simple as the global OGM building. In the global OGM building, the standard binary Bayes filter reported in [16] is widely used. Unfortunately, however, the standard binary Bayes filter cannot be applied directly to the local OGM building since all the static things in the environment seem to move due to the ego motion. Thus, to resolve the difficulty in the local OGM building, the dynamic binary Bayes filter developed in [17] is employed to build an OGM relative to the local vehicle coordinate in this paper. Furthermore, dynamic moving objects are detected by using a measure for segments and removed from the local OGM. In this paper, the map built relative to the local frame with dynamic objects removed is referred to as Static Local Coordinate Occupancy Grid Map (SLOGM) and it is used as a feature for the intersection type recognition. Finally, the nearest neighbor (NN) classifier with the proposed similarity is applied to the intersection type recognition.

This paper is organized as follows: in Section 2, the detail algorithm for building SLOGM is described. In Section 3, new intersections type recognition method using SLOGM is developed. The experimental results of the proposed method are presented in Section 4. Finally, some conclusions are drawn in Section 5.

## 2. Static Local Coordinate Occupancy Grid Map

### 2.1. Motivation

The OGM provides a very reliable framework to represent an environment and the OGM is widely used in robotics to represent a map [12,13,14,15]. Unfortunately, however, the standard OGM built relative to the global coordinates has a drawback: Memory requirements for the implementation of the OGM are extremely high when the OGM covers a spacious region. Thus, the OGM can be applied to only small or medium sized indoor environments such as a room, or a building, etc., and the OGM is restricted from the use in spacious outdoor roads. The drawback prevents the OGM from being used as a feature for the intersection type recognition.

To cope with the above problems, *the local OGM* is developed in this paper. The local OGM developed herein is defined not relative to the global coordinate but relative to the local vehicle coordinate in this paper. The local OGM building, however, is not as simple as the global OGM building. The standard binary Bayes filter reported in [16] is widely used in the global OGM building, but it cannot be used in a straightforward manner in the local OGM building. In the standard binary Bayes filter, *the environment is assumed not to change at all*. The assumption makes sense in the global OGM building, but it is not the case at all in the local OGM building because of the ego motion. More specifically, the environment *m* of interest is decomposed into a set of evenly spaced cells

(1) $$m_{t}=\left\{m_{t}^{1},m_{t}^{2},\cdots ,m_{t}^{N}\right\}$$

in the OGM, and each cell is allowed to take on either occupied (*O*) or free (*F*),

(2) $$m_{t}^{i}\in \left\{O,F\right\}$$

where the subscript *t* denotes the time; and *N* denotes the number of cells in the environment *m*. In the OGM building, the map is defined as a posterior probability $p(m_{t}^{i}|u_{1:t},z_{1:t})$ for each cell $m_{t}^{i}$, where $z_{1:t}=\left\{z_{1},z_{2},z_{3},\cdots ,z_{t}\right\}$ is the set of the laser scanner measurements corresponding to $m^{i}$ collected from time 1 to *t* and $u_{1:t}=\left\{u_{1},u_{2},u_{3},\cdots ,u_{t}\right\}$ is the set of control update corresponding to vehicle’s odometry collected from time 1 to *t*. In the global OGM building, $m_{t}^{i}$ is assumed to be unknown but fixed. That is,

(3) $$p(m_{t}^{i}=O|m_{t-1}^{i}=F)=p(m_{t}^{i}=F|m_{t-1}^{i}=O)=0$$

and

(4) $$p(m_{t}^{i}=O|m_{t-1}^{i}=O)=p(m_{t}^{i}=F|m_{t-1}^{i}=F)=1$$

**Figure 1:**
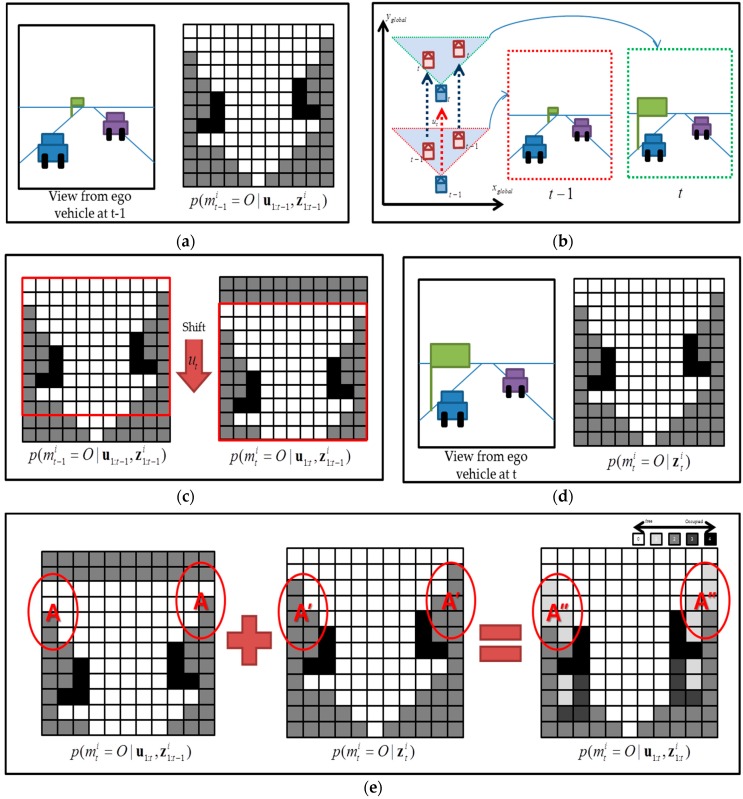
The illustration of incomplete OGM update using standard binary Bayes filter in local coordinate system. (**a**) The local OGM, which is built at t−1 $p(m_{t-1}^{i}=O|u_{1:t-1},z_{1:t-1}^{i})$; (**b**) Ego vehicle moves forward with two preceding vehicles at the same speed; (**c**) The local OGM is shifted downwards to turn $p(m_{t}^{i}=O|u_{1:t},z_{1:t-1}^{i})$ using the vehicle’s odometry; (**d**) The new measurement (inverse) likelihood OGM $p(m_{t}^{i}=O|z_{t}^{i})$; and (**e**) The updated OGM $p(m_{1:t}|u_{1:t},z_{1:t})$. The red circle region A’’ indicates the drawback of the static binary filter.

Thus, if $m_{t}^{i}$ is changed, it takes a long time for $p(m_{t}^{i}|u_{1:t},z_{1:t})$ to change from zero to one (or one to zero). Thus, the direct application of the standard Bayes filter to *the local OGM building* causes the problem, as shown in Figure 1. Let us suppose that the autonomous vehicle is driving along the highway with two preceding vehicles at time t−1 as in Figure 1a. The two preceding vehicles drive at the same speed as the autonomous vehicle.

The first figure in Figure 1a is the view from the autonomous vehicle at time t−1 and the second figure in Figure 1a is the corresponding local OGM $p(m_{t-1}^{i}=O|u_{1:t-1},z_{1:t-1}^{i})$. At the next time t, the autonomous vehicle moves forward with two preceding vehicles at the same speed, as shown in Figure 1b. The view from the autonomous vehicle will change as shown in the Figure 1b. In order to build the local OGM relative to the autonomous vehicle, the previous local OGM $p(m_{t-1}^{i}=O|u_{1:t-1},z_{1:t-1}^{i})$ is shifted downwards according to the odometry of the autonomous vehicle and it turns out to be $p(m_{t}^{i}=O|u_{1:t},z_{1:t-1}^{i})$, as shown in Figure 1c. This step corresponds to the prediction step in Bayes filtering. At time t, the view from the ego vehicle will look like the first figure in Figure 1d and the sensor measurements $p(m_{t}^{i}=O|z_{t}^{i})$ as shown in the second figure in Figure 1d are presented. In the standard Bayes filter, the two OGMs are combined by Bayes rule as shown in Figure 1e. In the figure, a big “+” denotes the Bayes inference. In the inference, $p(m_{t}^{i}=O|u_{1:t},z_{1:t-1}^{i})$ in Figure 1c plays the role of a prior while $p(m_{t}^{i}=O|z_{t}^{i})$ in Figure 1d plays the role of a likelihood (more precisely, an inverse likelihood). In Figure 1e, the regions A and A’ correspond to the region A’’. In the prior $p(m_{t}^{i}=O|u_{1:t},z_{1:t-1}^{i})$, the region A is free since it *was* free in $p(m_{1:t-1}|u_{1:t-1},z_{1:t-1})$.

When the measurement $p(m_{t}^{i}=O|z_{t}^{i})$ is presented, the region A’ is not observed anymore because it is behind the preceding vehicles. By combining A and A’ by Bayesian inference, the region A’’ remains almost free, which is indicated in almost white color, as shown in Figure 1e, but, obviously, it is not the true. The region A’’ is unknown and should be marked in gray. The region A’’ will turn gray, but it will take some time.

Thus, to resolve the difficulty in the local OGM building, the dynamic binary Bayes filter developed in [17] is employed to build an OGM relative to the local vehicle coordinate in this paper. In the dynamic binary Bayes filter, the value of the cell in the OGM is assumed to change.

### 2.2. Occupancy Grid Mapping Relative to Autonomous Vehicles

In this paper, the dynamic binary Bayes filter developed in [17] is used to update the posterior $p(m_{t-1}^{i}|u_{1:t-1},z_{1:t-1}^{i})$ when a new measurement $z_{t}^{i}$ and new movement $u_{t}$ are presented. Obviously, each cell satisfies

(5) $$p(m_{t}^{i}=F|u_{1:t},z^{i}{}_{1:t})+p(m_{t}^{i}=O|u_{1:t},z^{i}{}_{1:t})=1$$

In the above equation, the posterior $p(m_{t}^{i}|u_{1:t},z_{1:t}^{i})$ can be rewritten into

(6) $$\begin{array}{ll} p(m_{t}^{i}|u_{1:t},z_{1:t}^{i}) & =p(m_{t}^{i}|u_{1:t},z_{t}^{i},z_{1:t-1}^{i})\\ & =\frac{p(z_{t}^{i}|m_{t}^{i},u_{1:t},z_{1:t-1}^{i})p(m_{t}^{i}|u_{1:t},z_{1:t-1}^{i})}{p(z_{t}^{i}|u_{1:t},z_{1:t-1}^{i})} \end{array}$$

Since the new measurement $z_{t}^{i}$ is independent of the previous measurements $z_{1:t-1}^{i}$ and movements $u_{1:t}$, we have

(7) $$p(z_{t}^{i}|m_{t}^{i},u_{1:t},z_{1:t-1}^{i})=p(z_{t}^{i}|m_{t}^{i})$$

Thus,

(8) $$p(m_{t}^{i}|u_{1:t},z_{1:t}^{i})=\frac{p(z_{t}^{i}|m_{t}^{i})p(m_{t}^{i}|u_{1:t},z_{1:t-1}^{i})}{p(z_{t}^{t}|u_{1:t},z_{1:t-1}^{i})}$$

Applying the Bayes rule to the likelihood term $p(z_{i}^{t}|m_{t}^{i})$ in Equation (8) yields,

(9) $$p(m_{t}^{i}|u_{1:t},z_{1:t}^{i})=\frac{p(m_{t}^{i}|z_{t}^{i})p(z_{t}^{i})}{p(m_{t}^{i})}\frac{p(m_{t}^{i}|u_{1:t},z_{1:t-1}^{i})}{P(z_{t}^{i}|u_{1:t},z_{1:t-1}^{i})}$$

Thus, the posterior can be computed by

(10) $$p(m_{t}^{i}=O|u_{1:t},z_{1:t}^{i})=\frac{p(m_{t}^{i}=O|z_{t}^{i})p(z_{t}^{i})}{p(m_{t}^{i}=O)}\frac{p(m_{t}^{i}=O|u_{1:t},z_{1:t-1}^{i})}{P(z_{t}^{i}|u_{1:t},z_{1:t-1}^{i})}$$

and

(11) $$p(m_{t}^{i}=F|u_{1:t},z_{1:t}^{i})=\frac{p(m_{t}^{i}=F|z_{t}^{i})p(z_{t}^{i})}{p(m_{t}^{i}=F)}\frac{p(m_{t}^{i}=F|u_{1:t},z_{1:t-1}^{i})}{P(z_{t}^{i}|u_{1:t},z_{1:t-1}^{i})}$$

Dividing the above two equations yields:

(12) $$\frac{p(m_{t}^{i}=O|u_{1:t},z_{1:t}^{i})}{p(m_{t}^{i}=F|u_{1:t},z_{1:t}^{i})}=\frac{p(m_{t}^{i}=O|z_{t}^{i})}{p(m_{t}^{i}=F|z_{t}^{i})}\frac{p(m_{t}^{i}=F)}{p(m_{t}^{i}=O)}\frac{p(m_{t}^{i}=O|u_{1:t},z_{1:t-1}^{i})}{p(m_{t}^{i}=F|u_{1:t},z_{1:t-1}^{i})}$$

Applying the total probability theorem and Markov assumption:

(13) $$\begin{array}{l} \frac{p(m_{t}^{i}=O|u_{1:t},z_{1:t}^{i})}{p(m_{t}^{i}=F|u_{1:t},z_{1:t}^{i})}\\ =\frac{p(m_{t}^{i}=O|z_{t}^{i})}{p(m_{t}^{i}=F|z_{t}^{i})}\frac{p(m_{t}^{i}=F)}{p(m_{t}^{i}=O)}\\ \;\times \frac{p(m_{t}^{i}=O|m_{t-1}^{i}=O)p(m_{t-1}^{i}=O|u_{1:t},z_{1:t-1}^{i})+p(m_{t}^{i}=O|m_{t-1}^{i}=F)p(m_{t-1}^{i}=F|u_{1:t},z_{1:t-1}^{i})}{p(m_{t}^{i}=F|m_{t-1}^{i}=O)p(m_{t-1}^{i}=O|u_{1:t},z_{1:t-1}^{i})+p(m_{t}^{i}=F|m_{t-1}^{i}=F)p(m_{t-1}^{i}=F|u_{1:t},z_{1:t-1}^{i})} \end{array}$$

For simplicity, we assume that the state transition probability is constant, and then the state transition can be represented by the following four parameters

(14) $$\begin{array}{l} \pi_{11}=p(m_{t}^{i}=O|m_{t-1}^{i}=O)\\ \pi_{12}=p(m_{t}^{i}=O|m_{t-1}^{i}=F)\\ \pi_{21}=p(m_{t}^{i}=F|m_{t-1}^{i}=O)\\ \pi_{22}=p(m_{t}^{i}=F|m_{t-1}^{i}=F) \end{array}$$

where $\pi_{11}+\pi_{12}=1$ and $\pi_{21}+\pi_{22}=1$. Rearranging Equation (13) using simple two equations

(15) $$\begin{array}{l} (\pi_{11}+\pi_{21})\left(p(m_{t-1}^{i}=O|u_{t},z_{1:t-1}^{i})+p(m_{t-1}^{i}=F|u_{t},z_{1:t-1}^{i})\right)=1\\ (\pi_{21}+\pi_{22})\left(p(m_{t-1}^{i}=O|u_{t},z_{1:t-1}^{i})+p(m_{t-1}^{i}=F|u_{t},z_{1:t-1}^{i})\right)=1 \end{array}$$

then,

(16) $$\begin{array}{l} \frac{p(m_{t}^{i}=O|u_{1:t},z_{1:t}^{i})}{p(m_{t}^{i}=F|u_{1:t},z_{1:t}^{i})}\\ =\frac{p(m_{t}^{i}=O|z_{t}^{i})}{1-p(m_{t}^{i}=O|z_{t}^{i})}\frac{1-p(m_{t}^{i}=O)}{p(m_{t}^{i}=O)}\frac{\pi_{11}p(m_{t-1}^{i}=O|u_{1:t},z_{t-1}^{i})+\pi_{12}(1-p(m_{t-1}^{i}=O|u_{1:t},z_{t-1}^{i}))}{1-\left(\pi_{11}p(m_{t-1}^{i}=O|u_{1:t},z_{t-1}^{i})+\pi_{12}(1-p(m_{t-1}^{i}=O|u_{1:t},z_{t-1}^{i}))\right)}\\ =\frac{p(m_{t}^{i}=O|z_{t}^{i})}{1-p(m_{t}^{i}=O|z_{t}^{i})}\frac{1-p(m_{t}^{i}=O)}{p(m_{t}^{i}=O)}\frac{1-\left(\pi_{21}p(m_{t-1}^{i}=O|u_{1:t},z_{t-1}^{i})+\pi_{22}(1-p(m_{t-1}^{i}=O)|u_{1:t},z_{t-1}^{i})\right)}{\pi_{21}p(m_{t-1}^{i}=O|u_{1:t},z_{t-1}^{i})+\pi_{22}(1-p(m_{t-1}^{i}=O|u_{1:t},z_{t-1}^{i}))}\\ =\rho_{t}^{i} \end{array}$$

Finally, the modified binary Bayes filter for local OGM is,

(17) $$p(m_{t}^{i}=O|u_{1:t},z_{1:t}^{i})=\frac{\rho_{t}^{i}}{1+\rho_{t}^{i}}$$

**Figure 2:**
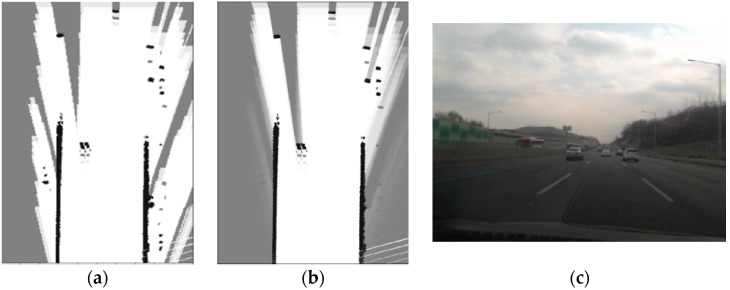
(**a**) Result of static binary Bayes filters; (**b**) Result of dynamic binary Bayes filters; and (**c**) The real world image (highway).

In Figure 2, the static and dynamic binary Bayes filters are applied to the real sensor data and the local OGM is built relative to the vehicle coordinate. Figure 2a,b are the results of static and dynamic binary filters, respectively, and Figure 2c is the corresponding world image taken on a highway. In the Figures, the darker the cell is, the more likely occupied the corresponding cell is. When the static method developed in [16] is used, the problem explained in Figure 1 arises, as shown in Figure 2a. The regions occluded by guardrail have bright cells, and it means that these cells are unoccupied, but it is not true. Unlike the static method [16], however, when the dynamic filter is applied, the occluded region behind the guardrail has occupancy probabilities, which correspond to unknown regions, as shown in Figure 2b.

### 2.3. Dynamic Object Removal

**Figure 3:**
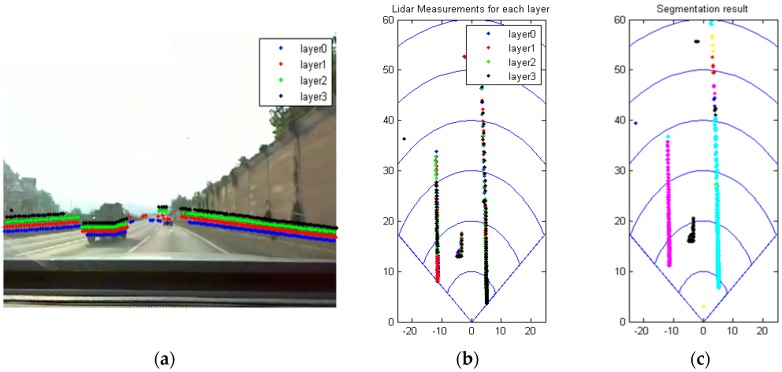
(**a**) The camera image superimposed with raw laser scanner data; (**b**) Raw laser scanner data. Layer 0 (**blue**), layer 1 (**red**), layer 2 (**green**), and layer 3 (**black**); and (**c**) The segmentations on the grid map. Different color means that different segments.

**Figure 4:**
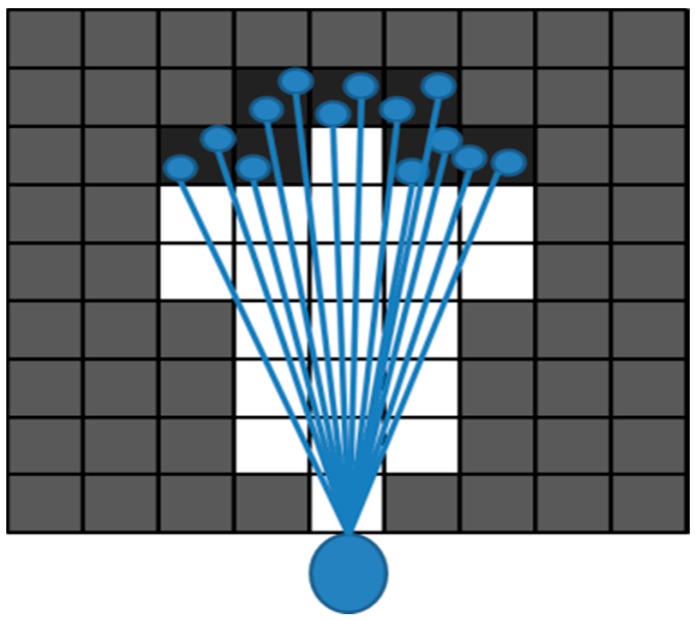
The illustration of difference between $z_{t}^{i}$ and ${\hat{z}}_{t}^{j}$. The $z_{t}^{i}$ indicates the set of laser beams, and ${\hat{z}}_{t}^{j}$ indicates the set of cells in the occupancy grid map which were hit by laser beams. The gray cells are the unknown region, and the set of dark cells is one segment, which is segmented as an independent object.

There might be a number of moving objects on the intersections, and they are likely to interfere with the intersection type recognition. Thus, the dynamic objects are removed before the intersection type recognition in this paper. A typical example is given in Figure 3. Figure 3a,b depict the raw measurements from the scanner, while Figure 3c depicts the segmentation results obtained by the ABD (adaptive breakpoints detection) [18]. In Figure 3a,b, the colors indicate layer information. Layer 0, 1, 2 and 3 are indicated in blue, red, green and black, respectively. In Figure 3c, different colors mean different segments. Three large segments are formed, and they are indicated in magenta, cyan and black. The three segments correspond to the left and right road boundaries and the preceding car, respectively. The black segment should be removed before the intersection type recognition. Let us denote the segment at time t by

(18) $${\hat{z}}_{t}^{j}=\left\{{\hat{z}}_{t}^{1,M_{1}},{\hat{z}}_{t}^{2,M_{2}},{\hat{z}}_{t}^{3,M_{3}},\cdots ,{\hat{z}}_{t}^{j,M_{j}}\right\},\;j=1,\cdots ,N_{t}$$

where j is the index for the segments and $N_{t}$ denotes the number of the segments formed at time t. The index $M_{j}$ denotes the length of the jth segment ${\hat{z}}_{t}^{j}$. The difference between $z_{t}^{j}$ and ${\hat{z}}_{t}^{j}$ is explained in Figure 4. Then, let us denote the region in the local OGM $m_{t}$, which is hit by ${\hat{z}}_{t}^{j}$ as

(19) $${\hat{m}}_{t}^{j}=\left\{m_{t}^{i}\in m_{t}|pos\left(m_{t}^{i}\right)=pos\left({\hat{z}}_{t}^{j,p}\right),i=1,\cdots ,N,p=1,\cdots ,M^{j}\right\}$$

where $pos\left(\cdot \right)\in {ℜ}^{2}$ denotes the longitudinal and latitudinal coordinates relative to the autonomous vehicle. To choose the dynamic segments which move over time, a new score,

(20) $$S\left({\hat{z}}_{t}^{j}\right)=1-\frac{\text{card}\left({\hat{m}}_{t}^{j}\cap {\hat{m}}_{t-1}^{j}\right)}{\text{card}\left({\hat{m}}_{t}^{j}\right)}$$

is defined to measure the degree to which the segment ${\hat{z}}_{t}^{j}$ can be classified as a dynamic object, where card(⋅) denotes the cardinality of the argument set. The physical meaning of this score is that the less the intersection between ${\hat{m}}_{t}^{j}$ and ${\hat{m}}_{t-1}^{j}$, the larger the score is, and the more likely the segment comes from a dynamic object.

**Figure 5:**
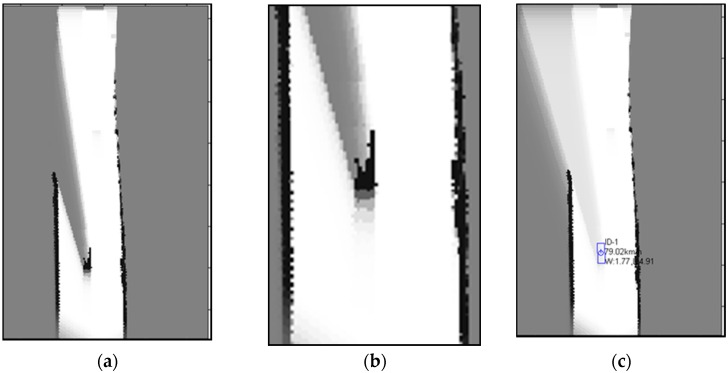
(**a**) The grid map with dynamic object; (**b**) The trails of dynamic segment; and (**c**) The static grid map with dynamic object removed. The **blue** box means the dynamic object information.

In conclusion, if

(21) $$S\left({\hat{z}}_{t}^{j}\right)>\delta$$

then ${\hat{m}}_{t}^{j}$ is quite different from ${\hat{m}}_{t-1}^{j}$ and the corresponding segment ${\hat{z}}_{t}^{j}$ is classified as a dynamic object, where δ is a threshold for the dynamic object. If

(22) $$S\left({\hat{z}}_{t}^{j}\right)\leq \delta$$

then the corresponding segment ${\hat{z}}_{t}^{j}$ does not move much, and it is classified as a static object. The result of the dynamic object removal is shown in Figure 5. The local OGMs before and after removing the dynamic object are depicted in Figure 5a,c respectively, and the segment corresponding to the preceding vehicle is magnified in Figure 5b. As shown in the Figures, a preceding vehicle with gray trail is present, but the vehicle is finally removed in Figure 5c. From now on, we call the OGM without dynamic object in autonomous vehicles coordinate as static local coordinate occupancy grid map, SLOGM.

## 3. Intersection Type Recognition Using the SLOGM

In this section, a new intersection type recognition method is presented. It is actually a multiple-class classification problem and the SLOGM explained in Section 2 is used as a feature for the classification problem. For the sake of simplicity, a new similarity measure is developed and a nearest neighbor (NN) classifier is used based on the new similarity measure.

### 3.1. Intersection Types

**Figure 6:**
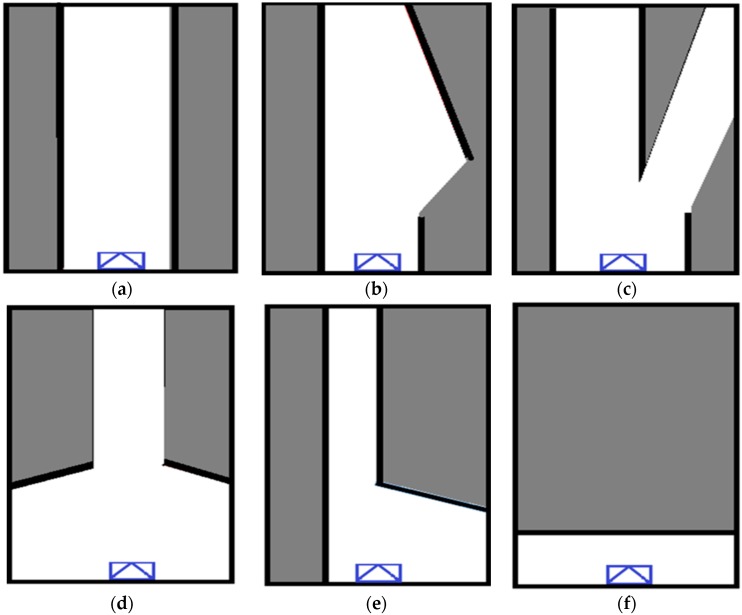
These are simplified shapes of the grid map represented as graphically; (**a**) Highway more than two lanes; (**b**) Merge-roads; (**c**) Diverge-roads; (**d**) Plus-shape intersections; (**e**) First type T-shape junctions; (**f**) Second type T-shape junctions.

In this paper, six types of intersections are considered and they are a highway, a merge-road, a diverge-road, a plus-shaped intersection and two kinds of T-shaped junctions as shown in Figure 6a through Figure 6f, respectively. Thus, the problem considered herein is a six-class classification problem, and the SLOGM is used as a feature for the classification.

The highway is a simple straight road and it looks like ‘I’ as shown in Figure 6a. The merge-road is a junction road at which additional road joins the main road, and, thus, it looks like an upside down lower case “y” as shown in Figure 6b. The diverge-road is a junction road at which the main road splits into two roads, and it looks like “y” as shown in Figure 6c. At a plus-shaped intersection, two roads meet and cross each other as shown in Figure 6d. The current longitudinal road is clearly observed by the laser scanner, but the two latitudinal roads are only partially observed due to the limited field of view (FOV) of the laser scanner. At the first type T junction, the current longitudinal road is merged with another latitudinal road at the right angle, as shown in Figure 6e. At the second type T junction, the current longitudinal road ends and the vehicles can go either to the left or to the right in the perpendicular direction, as shown in Figure 6f. Thus, a total of six classes are considered in this paper.

### 3.2. New Similarity Measure and Nearest Neighbor Classifier

In this subsection, a new similarity measure using SLOGM is developed for the intersection type recognition, and it is applied to implement the NN classifier. Let us suppose that we are given a training set D with $N_{train}$ training samples

(23) $$D=\left\{\left(M_{1},t_{1}\right),\left(M_{2},t_{2}\right),\left(M_{3},t_{3}\right),\cdots ,\left(M_{N_{train}},t_{N_{train}}\right)\right\}$$

where $M_{n}={\left[\begin{array}{cccc} M_{n}^{1} & M_{n}^{2} & \cdots & M_{n}^{N} \end{array}\right]}^{T}\in {\left[0,1\right]}^{N}$ is the SLOGM with the size N and $t_{n}\in \left\{H,M,D,P,T_{1},T_{2}\right\}$ is the associated intersection type; $n=1,2,\cdots ,N_{train}$ is an index for training samples. Here, $H,M,D,P,T_{1}$ and $T_{2}$ mean ‘highway’, ‘merge road’, ‘diverge road’, ‘Plus-shape intersections’, ‘first type T-junction’, and ‘second type T-junction’, respectively. To apply the NN to the intersection type recognition, a new similarity measure is developed. When two SLOGMs L and M are given and $L,M\in {\left[0,1\right]}^{N}$, the overlapped area (*OA*) between them is defined based on their free space by

(24) OA(L,M)=card{Free(L)∩Free(M)}

where $Free(M)=\left\{i|M^{i}<\varepsilon_{free},i=1,2,\cdots ,N\right\}$is a set of cells in SLOGM, which has low occupancy probability and corresponds to the drivable roads; $\varepsilon_{free}$ is a small threshold to determine whether a cell is free or occupied and card(⋅) denotes a cardinality of a set. That is, the *OA* implies the degree to which two SLOGMs share the drivable roads. Then, the similarity between the two SLOGMs L and M is defined by

(25) $$SIM\left(L,M\right)=\frac{OA^{2}\left(L,M\right)}{Free(L)\times Free(M)}\in \left[0,1\right]$$

The similarity measure SIM(L,M) is actually the squared geometric mean of *normalized overlapped area* between two SLOGMs and when two SLOGMs have the similar drivable roads, SIM(L,M) becomes close to 1. When a training set D in Equation (23) is given and a test SLOGM $L\in {\left[0,1\right]}^{N}$ is presented, the intersection type of the SLOGM M can be predicted by an NN classifier by

(26) $$Type\;of\;M=t_{n}\in \left\{H,M,D,P,T_{1},T_{2}\right\}$$

where $t_{NN}=\underset{n}{\arg\max}\left\{SIM(L,M_{n})\right\}$.

## 4. Experiment Setup

**Figure 7:**
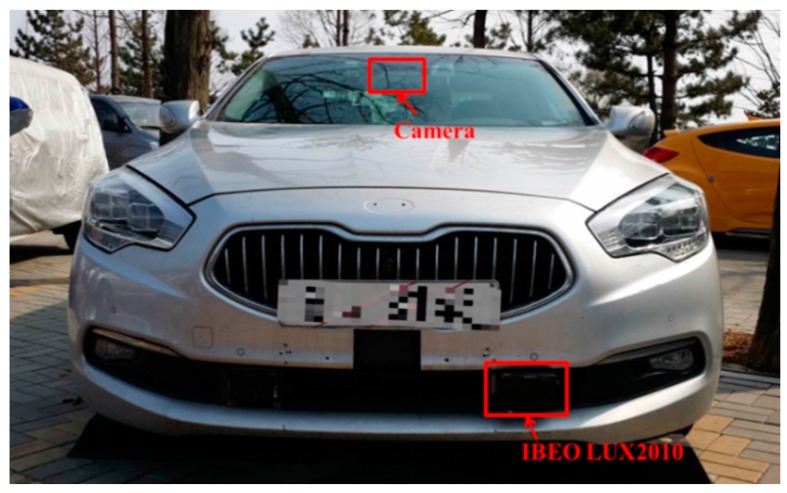
Vehicle equipped with a multi-layer laser scanner and a camera [18].

The validity of the proposed method is demonstrated through experimentation. The LUX2010 of IBEO (Hamburg, Germany) is used as a multi-layer laser scanner and it is installed on the KIA K5 (Seoul, Korea) as shown in Figure 7. The horizontal FOV of the LUX2010 is 110 degrees with 0.125 degree resolutions and vertical FOV is 3.2 degree with 0.8 resolutions. A single camera is also installed on the top of the windshield to gather the ground truth (GT) of the intersection types. A total of 1213 SLOGM samples are collected and each class has the similar number of samples. Each SLOGM sample covers the area of 80 m×50 m and each cell in SLOGMs is 0.25 m×0.25 m large.

To show the validity of the proposed method, its performance is compared with that of [6,8]. In this experiment, [6,8] were implemented by the authors. The previous two works used the 3D scanner with 32 or 64 layers, respectively, but we implemented their ideas using the 2D laser scanner IBEO LUX 2010 with four layers for fair comparison with the proposed method.

**Figure 8:**
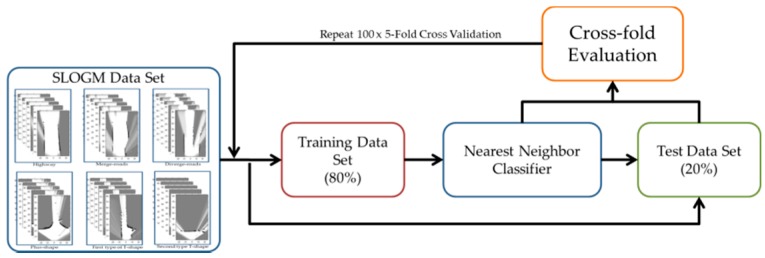
Outline of the experiment.

For the validation of the proposed system, five-fold cross validation is conducted. The whole set of samples is partitioned into five subsets with the same size in a random manner. The first four sets are used as a training set, and the last set is used as a test set as in Figure 8. Then, three of the first four sets and the last set are used as a training set, and the remaining set is used as a test set in turn. The similar process is repeated three more times such that all the five subsets are used as a test set exactly once. The five-fold cross validations are run 100 times, and the results are summarized in the next subsection.

## 5. Experiment Results

**Figure 9:**
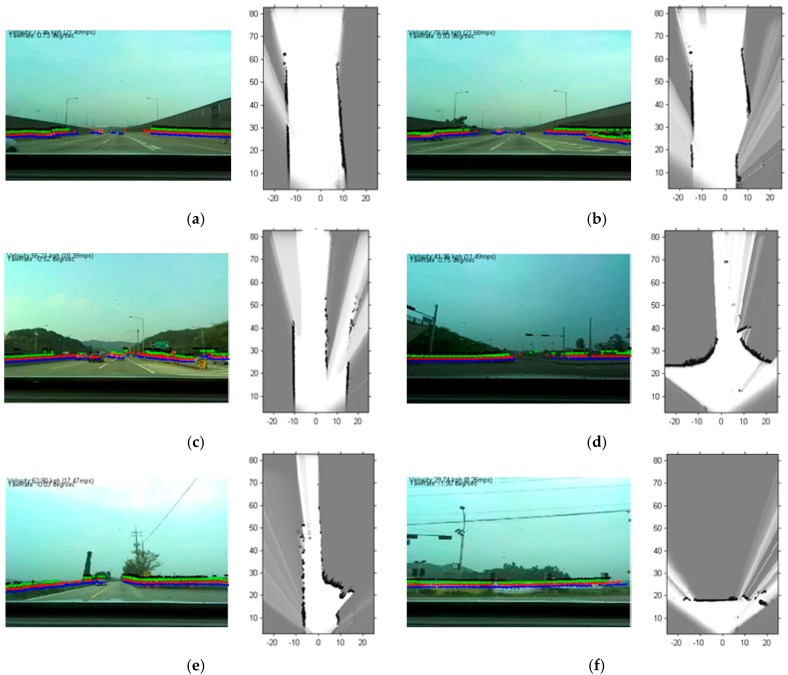
The results of intersections recognition. (**a**) Highway; (**b**) Merge-road; (**c**) Diverge-road; (**d**) Plus-shape intersections; (**e**) First type T-shape junctions; (**f**) Second type T-shape junctions; left side images are camera image with raw data calibration; right side images are static local coordinate occupancy grid map (SLOGM).

The NN classifier based on the proposed similarity is applied to the intersection type recognition. The six classes considered herein are summarized in Table 1 As stated, five-fold cross validation is conducted one hundred times and the quantitative results of intersection type recognition are summarized in Table 2, and some illustrative examples are given in Figure 9. Table 2 is the confusion matrix for the six classes. In Figure 9, the first and third columns show the actual environments superimposed with the laser scanner and the second and fourth columns are the corresponding SLOGMs.

As shown in Table 2, the class $T_{1}$ (the first type T-junction) demonstrates the highest true positive rate (TPR) among the six classes, and it is 91.575%. Following the class $T_{1}$, the class $T_{2}$ (second type T-shape junction) has the second highest TPR and it is 91.532%. The reason for the excellence of the two T-junctions is that their SLOGMs have relatively unique shapes from other SLOGMs. In class $T_{1}$ (first type T junction), a single latitudinal road joins the main longitudinal road at the junction in the perpendicular direction, as shown in Figure 9e. In class $T_{2}$ (second type T junction), the current main road ends and branches off in to the left or in to the right in the perpendicular direction, as shown in Figure 9f. The unique shape in the two junctions gives them high similarity scores and distinguishes the two sets of samples from the others. On the other hand, the class M (merge-road) is the one which is the most difficult to classify. Since the FOV of a laser scanner is limited, the whole intersection is rarely observed. When the autonomous vehicle enters a merge-road, the road might look like just a highway or a common straight road because the boundaries of the intersection block the laser scanner from scanning the latitudinal roads as shown in Figure 9b. For the similar reason, the class H is also hard to identify because it shares the common characteristic of *straightness* with other intersections.

**Figure 10:**
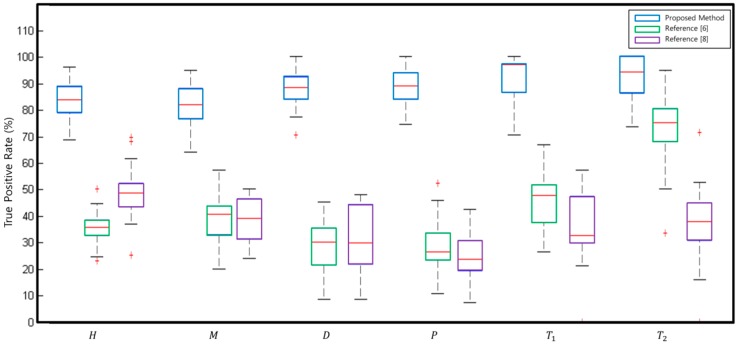
The box plot for each class.

Finally, the proposed method is compared with the previous works [6,8] in terms of true positive rate (TPR). The TPR of [6,8] reported herein are lower than the original values reported in [6,8] because the current TPR are obtained using the four layer IBEO LUX 2010, while the original TPR were obtained using the 3D Velodyne (Morgan Hill, CA, US) with 32 or 64 layers. A box plot is depicted to visualize the cross validation results as in Figure 10. From the figure, the proposed method outperforms the previous two methods. The average TPR of [6,8] are 54.61% and 46.91%, respectively, while that of the proposed method is 89.15%. The reason for the excellence of the proposed method might be that the SLOGM proposed herein is a good match with the 2D multi-layer laser scanner and has stronger discriminative power than the previous features of [6,8] for the intersection type recognition using a laser scanner.

## 6. Conclusions

In this paper, a new intersection type recognition method has been proposed. Unlike the previous works, the occupancy grid map was built relative to the local coordinate and the intersection type was recognized based on the local OGM. The dynamic binary Bayes filter was employed to solve the cell change problem which arises in the local OGM building. A new measure $S\left({\hat{z}}_{t}^{j}\right)$ was proposed to remove the moving dynamic objects from the local OGM. Furthermore, a new similarity measure SIM(L, M) between two SLOGMs was developed, and it was combined with an NN classifier to implement the intersection type recognition system. Finally, the proposed method was applied to a real world problem and its validity was verified by experimentation.

## Figures and Tables

**Table 1:** Types of intersections.

Class	Type of Intersections
H	Highways
M	Merge-roads
D	Diverge-roads
P	Plus-shape intersections
$T_{1}$	First type T-shape junctions
$T_{2}$	Second type T-shape junctions

**Table 2:** Confusion matrix.

	Prediction	H	M	D	P	$T_{1}$	$T_{2}$
Actual							
H		**82.76%**	4.01%	8.11%	2.75%	1.05%	1.33%
M		3.96%	**81.06%**	8.23%	1.63%	-	5.12%
D		2.29%	1.89%	**87.63%**	2.64%	1.15%	4.41%
P		-	-	0.24%	**87.43%**	0.54%	11.79%
$T_{1}$		-	-	-	-	**91.58%**	8.43%
$T_{2}$		-	-	-	5.53%	2.94%	**91.53%**
